# Salvage percutaneous coronary intervention for failed graft itself three days after minimally invasive direct coronary artery bypass

**DOI:** 10.1093/jscr/rjad420

**Published:** 2023-07-31

**Authors:** Masahiko Narita, Shingo Kunioka, Yuya Kitani, Tomonori Shirasaka, Toshiharu Takeuchi, Hiroyuki Kamiya

**Affiliations:** Department of Cardiac Surgery, Asahikawa Medical University, Asahikawa, Japan; Department of Cardiac Surgery, Asahikawa Medical University, Asahikawa, Japan; Division of Cardiology, Nephrology, Pulmonology, and Neurology, Department of Internal Medicine, Asahikawa Medical University, Asahikawa, Japan; Department of Cardiac Surgery, Asahikawa Medical University, Asahikawa, Japan; Division of Cardiology, Nephrology, Pulmonology, and Neurology, Department of Internal Medicine, Asahikawa Medical University, Asahikawa, Japan; Department of Cardiac Surgery, Asahikawa Medical University, Asahikawa, Japan

## Abstract

Minimally invasive direct coronary artery bypass is preferred due to its less invasive nature; however, it carries the risk of graft failure owing to inherent technical challenges. We present a case where minimally invasive direct coronary artery bypass grafting was performed and graft failure was detected via coronary angiography 3 days post-operation. Successful percutaneous coronary intervention was subsequently performed on the failed graft itself to salvage myocardial cellular damage. Consequently, the combination of minimally invasive direct coronary artery bypass and percutaneous coronary intervention, both less-invasive revascularization approaches, effectively achieved the primary treatment objective.

## INTRODUCTION

Minimally invasive direct coronary artery bypass (MIDCAB) grafting is recommended for high-risk patients as a less-invasive alternative to full sternotomy revascularization; however, it is associated with the risk of graft failure owing to its technically demanding nature [1]. Herein, we report a case in which early postoperative coronary angiography (CAG) detected graft failure following MIDCAB, and subsequent percutaneous coronary intervention (PCI) to the left internal thoracic artery (LITA) graft salvaged myocardial cellular damage.

## CASE REPORT

An 85-year-old male patient with a thoracic aortic aneurysm was admitted for thoracic endovascular aortic repair (TEVAR). Preoperative computed tomography (CT) showed diffusely calcified coronary arteries. However, because myocardial scintigraphy detected no ischemic manifestation and transthoracic echocardiography showed no wall motion abnormality, coronary artery intervention was deemed unnecessary. TEVAR was successfully performed; however, on postoperative Day 14, the patient complained of recurrent chest tightness. Electrocardiography showed slight ST elevation from V5 to V6 and slight ST depression in the inferior leads (Supplemental Fig. S1), and serum troponin I level was moderately elevated (108.3 pg/ml). CAG for suspected unstable angina revealed chronic total occlusion in the middle right coronary artery and middle left anterior descending artery (LAD), and 75–90% focal stenosis in the proximal circumflex branch (Supplemental Fig. S2). Considering the patient’s general profile, the cardiac team deemed isolated MIDCAB grafting on the LAD appropriate.

**Figure 1:**
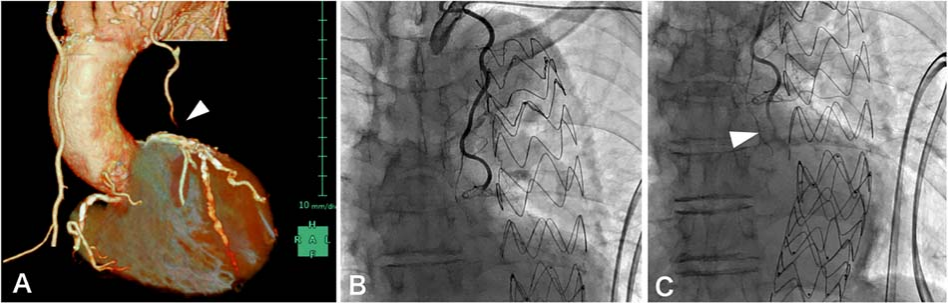
(A) contrast-enhanced MSCT on postoperative Day 3 showing peripheral LITA graft occlusion (arrowhead); (B, C) postoperative CAG demonstrated thrombotic occlusion in the peripheral LITA graft (arrowhead), which was considered resolvable by PCI.

**Figure 2:**
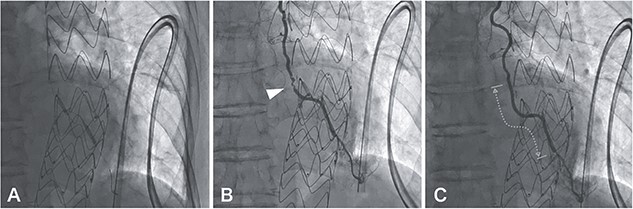
A 0.014-inch guidewire (SION Blue, Asahi Intecc, Aichi, Japan) was inserted into the LITA graft under intravascular ultrasound (Altaview; Terumo, Tokyo, Japan) guidewire; the diameter of the lesion was about 2 mm, therefore balloon dilation (Ryurei 2.0 × 20 mm, Terumo, Tokyo, Japan) was performed first from distal to proximal (A); angiography showing hematoma formation in the lesion, which was not resolved by the administration of nitroglycerin (B, arrowhead); three stents (Synergy 2.25 × 12 mm, 2.25 × 28 mm, 2.25 × 20 mm, Boston Scientific, USA) were overlappingly placed to cover the lesion (C, dotted line), and there was a satisfactory angiographic result.

MIDCAB, harvesting of a pedicled LITA followed by an end-to-side anastomosis to the LAD with a sufficient intraoperative graft flow, was performed through left anterior mini-thoracotomy. Although the patient’s chest symptoms were vague and serum cardiac biomarkers were not elevated postoperatively, routine contrast-enhanced multi-slice CT (MSCT) on postoperative Day 3 suggested the middle of LITA graft occlusion (Fig. 1A). The following CAG also demonstrated thrombotic occlusion (Fig. 1B and C). Subsequently, we performed PCI for the lesion in the LITA graft. The lesion was crossed with a 0.014-inch guidewire (SION Blue, Asahi Intecc, Aichi, Japan); afterward, balloon dilation (Ryurei 2.0 × 20 mm, Terumo, Tokyo, Japan) was performed repeatedly. Three stents (Synergy 2.25 × 12 mm, 2.25 × 28 mm, 2.25 × 20 mm; Boston Scientific, USA) were overlappingly placed to cover the lesion, and a satisfactory angiographic result was observed (Fig. 2). The patient had an unremarkable hospital stay and remained symptom-free 2 years postoperatively.

## DISCUSSION

To the best of our knowledge, this is the first reported case of a patient that underwent MIDCAB grafting and shortly after received successful PCI to a failed LITA graft; both less-invasive approaches achieved a primary treatment goal.

MIDCAB grafting is preferred because of its reduced invasiveness; however, an 8.9% risk of graft failure has been reported [1]. Perioperative graft failure following surgical revascularization may cause acute myocardial ischemia, resulting in increased mortality [2]. In the absence of uniform diagnostic criteria, postoperative CAG remains the gold standard for evaluating postoperative myocardial ischemia (PMI), and it may allow for subsequent PCI based on the clinical findings obtained by CAG [3]. Moreover, re-revascularization with rescue PCI may limit the extent of myocardial cellular damage compared with acute redo-CABG [2]. Thus, particularly for patients who prefer reduced invasiveness, it is reasonable to perform CAG for suspected PMI and consider the interventional approach as the first choice of treatment for early graft dysfunction.

While most previous reports have described the rescue PCI to lesions in the distal anastomosis site and native coronary [4–6], in this case, the target lesion location was the middle of the LITA graft. Considering the angiography findings, there was stenosis in the middle of the graft and the thrombus formation just proximal from it. Because the intraoperative graft flow was sufficient, structural dysfunction was unlikely, so the cause of graft stenosis was obscure. Graft intervention may be effective if the thrombus burden is not extensive [7]. Consequently, we performed PCI across the lesion in the graft and obtained a satisfactory result.

## Supplementary Material

MIDPCI_fs1_rjad420

MIDPCI_fs2_rjad420

## Data Availability

The authors confirm that the data suppo rting the findings of this study are availabl e within the article and its supplementary materials.
