# Supplementary figures and images for: Salvage percutaneous coronary intervention for failed graft itself three days after minimally invasive direct coronary artery bypass
Source: J Surg Case Rep. 2023 Jul 31;2023(7):rjad420. doi: 10.1093/jscr/rjad420 (PMC10389685; doi:10.1093/jscr/rjad420)

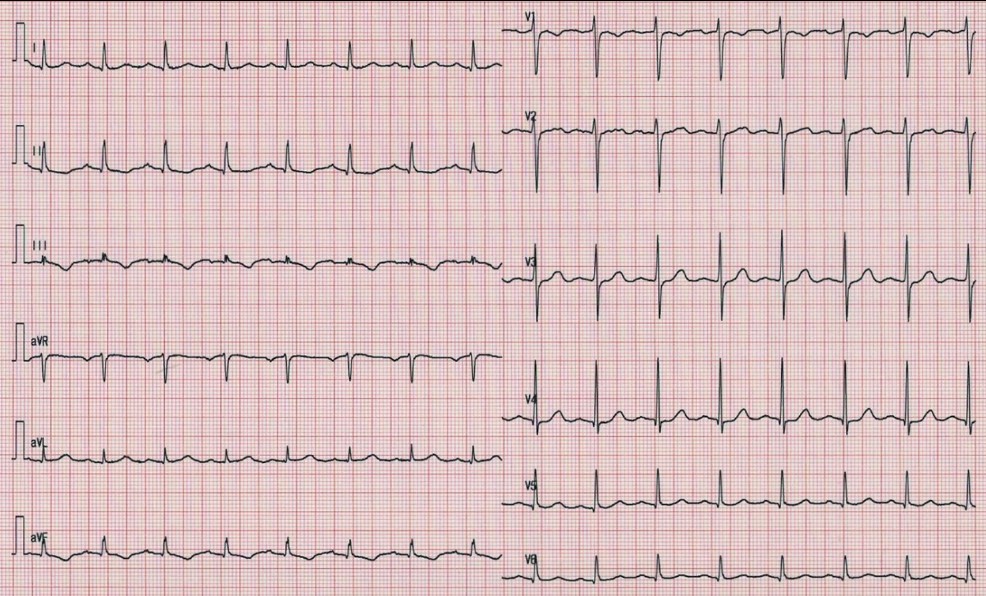

Supplement: MIDPCI_fs1_rjad420 [file midpci_fs1_rjad420.jpeg]

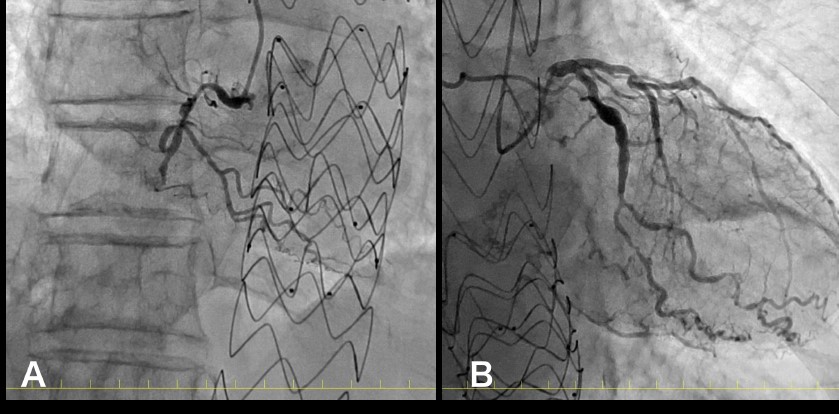

Supplement: MIDPCI_fs2_rjad420 [file midpci_fs2_rjad420.jpeg]
